# New *NR5A1* mutations and phenotypic variations of gonadal dysgenesis

**DOI:** 10.1371/journal.pone.0176720

**Published:** 2017-05-01

**Authors:** Ralf Werner, Isabel Mönig, Ralf Lünstedt, Lutz Wünsch, Christoph Thorns, Benedikt Reiz, Alexandra Krause, Karl Otfried Schwab, Gerhard Binder, Paul-Martin Holterhus, Olaf Hiort

**Affiliations:** 1 Department of Paediatrics and Adolescent Medicine, Division of Experimental Paediatric Endocrinology and Diabetes, University of Lübeck, Lübeck, Germany; 2 Department of Paediatric Surgery, University Hospital of Lübeck, Germany; 3 Department of Pathology, University Hospital of Lübeck, Lübeck, Germany; 4 Institute for Cardiogenetics, University of Lübeck, Lübeck, Germany; 5 Department of Paediatrics and Adolescent Medicine, Paediatric Endocrinology and Diabetes, University Hospital Freiburg, Freiburg, Germany; 6 Department of Paediatrics and Adolescent Medicine, Eberhard-Karls-University of Tübingen, Tübingen, Germany; 7 Division of Paediatric Endocrinology and Diabetes, Department of Paediatrics, Christian-Albrechts-University, Kiel, Germany; McGill University, CANADA

## Abstract

Mutations in *NR5A1* have been reported as a frequent cause of 46,XY disorders of sex development (DSD) associated to a broad phenotypic spectrum ranging from infertility, ambiguous genitalia, anorchia to gonadal dygenesis and female genitalia. Here we present the clinical follow up of four 46,XY DSD patients with three novel heterozygous mutations in the *NR5A1* gene leading to a p.T40P missense mutation and a p.^18^DKVSG^22^del nonframeshift deletion in the DNA-binding domain and a familiar p.Y211Tfs*83 frameshift mutation. Functional analysis of the missense and nonframeshift mutation revealed a deleterious character with loss of DNA-binding and transactivation capacity. Both, the mutations in the DNA-binding domain, as well as the familiar frameshift mutation are associated with highly variable endocrine values and phenotypic appearance. Phenotypes vary from males with spontaneous puberty, substantial testosterone production and possible fertility to females with and without Müllerian structures and primary amenorrhea. Exome sequencing of the sibling’s family revealed *TBX2* as a possible modifier of gonadal development in patients with *NR5A1* mutations.

## Introduction

The term “Disorders of sex development” (DSD) comprises a broad spectrum of clinical conditions affecting the gonadal and genital development. DSD is divided into three categories, namely numerical chromosomal abnormalities, 46,XX DSD and 46,XY DSD [1]. Various underlying causes have been described. Often it is not possible to determine a genotype-phenotype correlation due to the overlapping clinical presentations.

One gene emerging in the last years to be causative in 10–20% of 46,XY DSD is *NR5A1* encoding Steroidogenic factor-1 (SF-1) located on chr 9q33.3 (OMIM 184757) [2]. The nuclear receptor SF-1 plays a pivotal role in the adrenal and reproductive development and function as well as in transcription of genes involved in steroidogenesis [3, 4]. In early male development SF-1 is expressed in the bipotential gonad and regulates the differentiation towards testes through modulation of the expression of genes like *SRY* and *SOX9* [5, 6]. Besides, SF-1 is involved in the regression of the paramesonephric duct via initiation of the expression of Anti-Müllerian hormone (AMH) in Sertoli cells and the virilisation by regulation of the biosynthesis of testosterone in Leydig cells [7–9]. The majority of *NR5A1* mutations were described in 46,XY DSD patients. The phenotypical spectrum encompasses hypospadias [10–12], ambiguous genitalia like a hypoplastic phallus [13–15], or a complete external female appearance [16–19], but also only male infertility [20–22]. Furthermore, *NR5A1* mutations were found in 46,XX patients with premature ovarian failure and primary ovarian insufficiency [17, 23, 24]. Recently, three independent groups identified also a recurrent NR5A1 p.R92W mutation in several patients with 46,XX testicular/ ovotesticular DSD, highlighting the role of NR5A1 in the development of both testes and ovaries [25–27]. Except for five reported cases with adrenal insufficiency [28–32] and some patients with mild elevated ATCH [16, 33] most *NR5A1* mutations were described in 46,XY DSD patients with normal adrenal function at the date of examination.

Gonadotropins and sex hormone levels vary a lot in patients with *NR5A1* mutations. Beside 46,XY DSD patients with low concentrations of AMH and detectable Müllerian structures [14, 34] and patients with normal AMH levels without a uterus or fallopian tubes [15, 35], there are also some cases reported with low levels of AMH at birth but without apparent Müllerian structures [15, 36, 37]. Testosterone concentrations differ between patients with *NR5A1* mutations as well. In most cases testosterone is low in the neonatal period while the phenotype ranges from ambiguous genitalia to female external genitalia with or without well-developed Wolffian structures [37]. But there are also some patients reported having normal testosterone concentrations at birth and even at puberty which show a spontaneous pubertal progression [33, 38] or obvious virilisation [18]. Moreover, gonadal histology is also not consistent in these patients. Recent studies showed a pattern of focal aggregations of Leydig cells with cytoplasmic lipid droplets and a reduced number of thin seminiferous tubules in several young prepubertal patients, probably due to a reduced expression of steroidogenic acute regulatory protein (STAR) or CYP11A1 that was not described earlier [17, 39, 40]. This variability complicates the confirmation of a diagnosis and the assignment to one of the established categories of DSD, especially the discrimination from partial androgen insensitivity due to androgen receptor gene mutations [11, 15]. Here we describe the endocrine follow up and phenotypic variability of four 46,XY DSD patients and the functional characterization of their associated novel mutations in *NR5A1* and offer new explanations for phenotypic variation.

## Patients and methods

### Patients

Written informed consent was obtained from all patients and family members studied. The study was approved by the Ethical Committee of the University Hospital of Lübeck, Lübeck, Germany (AZ: 08–081).

#### Patient 1

Ambiguous genitalia were noted at birth and a thorough evaluation was initiated at the age of 4 weeks. Initially, a female sex assignment was favored. The clinical description noted normal weight (3250 g) and length (54 cm) and unsuspicious internal and neurological examination except for genital appearance. Prominent labioscrotal folds were noted with palpable gonads, the phallic structure measured 1.3 cm. The urethral and vaginal openings were not described. On urogenitoscopy, a normal bladder was seen, a small vagina, unclear uterine structures, but no colliculus seminalis. On laparoscopy, hypoplastic testicular appearing gonads were seen without Müllerian remnants. On histology, the gonads showed a neonatal testicular appearance with germ cells. Karyotype was 46,XY. Endocrine evaluation showed a high-normal ACTH of 54 pg/mL (reference < 60) with a cortisol of 26 ng/mL, which was interpreted as subnormal. LH was 3.9 IU/L, FSH 13.2 IU/L and interpreted as normal to slightly elevated (FSH reference < 11.0 IU/L), while testosterone was 20 ng/dL and seen also as subnormal. Endocrine testing was performed and revealed a normal response in the ACTH test with a rise in cortisol from 8.2 ng/mL to 54.3 ng/mL. On résumé, a male gender was favored and the child had been reassigned accordingly.

During childhood, several surgical procedures were performed to reconstruct male genital appearance including hypospadias repair, orchidopexy and scrotal fusion. At the age of 10;7 years, clinical appearance was described with a testicular volume of 3–4 cc on both sides and a Tanner stage G2. Hormonal evaluation showed LH 1.03 IU/L, FSH 8.8 IU/L and testosterone 82.6 ng/dL. Inhibin B was 19 ng/L (reference range of adult males 100–400). An hCG-Test was performed with 5.000 IU/m^2^ body surface area and after 72 hours, testosterone had increased to 242 ng/dL. This was seen as proper testosterone response, although the low inhibin B was seen as a possible failure of Sertoli cells. At the age of 11;3 years, inhibin B was below the detection limit, and at 11;11 years, FSH elevated to 20.9 IU/L, thus a progression of testicular dysfunction was presumed. At that time testosterone was 162 ng/dL with LH 5.2 IU/L. Therefore, additional testosterone gel therapy with initially 25 mg testosterone gel and later 37.5 mg was initiated to foster pubertal development. At the age of 15;8 years, the adolescent presented with a height of 180.5 cm, weight 64.3 kg. Genital appearance male according to Tanner stage 4, pubic hair Tanner stage 6. Testes were palpable with a volume of 4–6 cc. LH was 7.6 IU/L, FSH 18.2 IU/L, testosterone 508 ng/dL under supplementation with 37.5 mg testosterone gel. At this time, ACTH was 39.2 pg/mL, cortisol 154 ng/mL, which was interpreted as normal adrenal function and further molecular studies were initiated with the consent of the family. An ACTH-test with 250 μg Synacthen was performed at the age of 16 yrs. with basal cortisol at 182 ng/mL and 319 ng/mL after 60 minutes, thus corresponding with a good adrenal response. At the age of 18 years the patient had a low-normal testosterone production of 383 ng/dL (without testosterone supplementation), but increased gonadotropins (LH: 44.3 IU/L; FSH 56.1 IU/L). A spermiogram revealed azoospermia and the patient opted for a trial of testicular sperm extraction (TESE) and cryopreservation to preserve fertility.

#### Patient 2

The mother was a primipara and the parents are non-consanguineous. In the 14^th^ week of gestation, a thickened neck fold had been documented by routine ultrasound triggering subsequent chorionic villus biopsy. This excluded trisomy 21 and revealed a 46,XY karyotype. Unexpectedly, a second ultrasound in the 30^th^ week of gestation did not reveal evidence for male external genitalia. Delivery occurred in the 41^st^ week of gestation by emergency Caesarian section because of maternal fever due to urinary tract infection. External genitalia of the child were ambiguous with rugated labioscrotal folds, partial labioscrotal fusion and a micropenis of 1.3 cm in length thus confirming the prenatal ultrasound. Only in the left urogenital fold, a gonad of 0.5 cc was initially palpable. At the age of about 2 weeks, ultrasound revealed no evidence for an uterus and the 46,XY karyotype was confirmed. Basal androstenedione was 6 ng/dL (normal range 11.4–60 ng/dL), testosterone was < 1 ng/dL (normal range 2.8–362.9 ng/dL), and dihydrotestosterone was < 1 ng/dL (normal range 2.8–11.6 ng/dL). 72 hours following hGC stimulation with 1,200 IU (5,000 IU / m^2^), androstenedione rose to 40 ng/dL, testosterone to 8 ng/dL and DHT to < 1 ng/dL, demonstrating massively compromised gonadal androgen biosynthesis. LH was < 0.5 IU/L, FSH was 7.4 IU/L. No salt losing crises occurred in the newborn period or at any time later on. Plasma steroid profiling combined with a Synacthen test (125 μg) revealed the following basal and stimulated values (0 min / 60 minutes): cortisol 11 ng/mL (normal range 9.24–86.8 ng/mL) / 187ng/mL (normal range 54–718 ng/mL); aldosterone 0.28 ng/mL (normal range 0.14–1.05 ng/mL) / 0.45 ng/mL (normal range 0.25–20 ng/mL) demonstrating normal adrenal steroid biosynthesis. Further normal basal and stimulated values, respectively, were determined for progesterone, 11-deoxycorticosterone, corticosterone, 17OH-progesterone, and 11-deoxycortisol. All steroid hormones had been determined by multi-steroid RIA following extraction and chromatographic separation through Sephadex columns. Due to the presumed gonadal androgen biosynthesis defect in the absence of Müllerian structures and because the parents wished genetic counseling, molecular analyses of genes involved in gonadal androgen biosynthesis has been initiated. However, gene sequencing of *WT1*, *LHCGR* including exon 6A, *CYP17A1*, *HSD17B3* and *SRD5A2* revealed no mutations. In order to establish the DSD diagnosis, a combined examination by vaginoscopy and laparoscopy had been initiated. Vaginoscopy revealed a short urogenital sinus while laparoscopy confirmed the previous ultrasound findings demonstrating absence of Müllerian duct derivates. Two testis-like structures could be found in both inguinal channels but no epididymis and no ductus deferens was present on either side (S1 Fig). Additional analysis of the *NR5A1* gene had then been initiated. The child grew up in the female gender. The parents did only visit their general practitioner for routine check-ups in the meantime with no further appointments in the DSD outpatient clinic. At the age of 8;4 years, the general practitioner informed the DSD clinic that the child suffered from inguinal hernia. Gonadal biopsy revealed testicular tissue in both gonads with tubular structures, presence of Sertoli cells but no Leydig cells (S2 Fig). There was no indication for pre-malignant or malignant histological changes.

#### Patient 3

Patient 3 is the third child of healthy parents born at term with normal weight (3550 g) and length (50 cm). At birth a bipartite scrotum and a small phallus size of 20 mm length with chordae and scrotal hypospadias was noted. Both testes were descended and at day 2 a testosterone value of 187 ng/dL was measured. Abdominal sonography revealed no vagina or uterus. Subsequent chromosomal analysis revealed a normal 46,XY karyotype. Endocrine evaluation at 3.5 months revealed normal LH (2.6 IU/L) and FSH (3.7 IU/L) and inhibin B (144 ng/L) values. A stimulation test with hCG revealed normal basal/stimulated values for testosterone (232/735 ng/dL) and DHT (80/469 ng/dL). Hormone values for 17-hydroxpregnenolone (2257 ng/dL), DHEA (116 ng/mL), androstenedione (90 ng/mL), and hydroxyprogesterone (185 ng/dL) were unremarkable. Sex assignment was discussed with the parents and a male sex assignment was favored and the child received hypospadias repair. Sequence analysis of the *AR* gene revealed a short glycine repeat associated with micropenis and hypospadias [41] and partial androgen insensitivity syndrome (PAIS) was suspected. At the age of 12 years, a re-evaluation was performed. He had a short penis with a diameter of 1.5 cm. The testes were in the scrotum with a volume of 4 cc on the left side and 2 cc on the right side. Laboratory investigation revealed an LH of 2.4 IU/L (within the reference range), an FSH of 9 IU/L and a testosterone of 119 ng/dL which is in the reference range of pubertal stage Tanner 3. The inhibin B was much lower at this time with 36.2 pg/mL. At the age of 14;7 years, he had clinically advanced to pubertal stage Tanner PH 4 and G 4. The testicular volume was 6 cc and 8 cc, respectively. ACTH was 69.4 pg/mL (reference range < 63.1), Cortisol was 170 ng/mL and seen as unremarkable. LH was 3.4 IU/L, FSH was 16.2 IU/L (reference range < 12.4) and testosterone with 297 ng/dL in the reference range of pubertal stage Tanner 4. At the age of 16 years, the clinical appearance had not changed. ACTH was 45.9 pg/mL, cortisol 96 ng/mL, LH 7.5 IU/L, and testosterone 344 ng/dL. However, the FSH had risen to 27.1 IU/L and inhibin B decreased to 15 ng/L, which was interpreted as progressive failure of spermatogenesis. A sperm analysis was offered, but declined at this time.

#### Patient 4

Patient 4 is the elder sister of P3 and came to attention in our clinic because of primary amenorrhea at the age of 14;5 years. She presented without pubic hair or breast development (pubertal stage Tanner PH1/B1). A 46,XY karyotype was detected. LH (31.6 IU/L) and FSH (90.7 IU/L) values were highly elevated and testosterone (16 ng/dL) and inhibin B (<10 ng/L) values very low or even below the detection level. ACTH was 25.16 pg/mL and cortisol was 38.5 ng/mL both values within the normal reference ranges. On ultrasound a very small uterus of prepubertal shape and size was suspected. A laparoscopy was performed, which confirmed the presence of a small uterus and small dysgenetic gonads. The gonadal remnants were removed. The histology revealed rudimentary testicular tissue with fibrosis, some ducts and also Leydig cells. Also epididymal tissue and tubular tissue was identified (S3 Fig).

Puberty was induced with estradiol. At the age of 15;10 years, she received 2 mg of estradiol valerate. Breast development corresponded to pubertal stage Tanner 4. The uterus was now 4 cm long (S4 Fig). Gonadotropins were again highly elevated with LH at 49.3 IU/L and FSH 81.4 IU/L, and estradiol at 28.5 ng/L in the lower female reference range. The patient reported weak but regular menses under estrogen replacement.

The AR mutation previously detected in her brother was unlikely the reason for the observed phenotype of gonadal dysgenesis and a search for the genetic cause of the familiar DSD by exome sequencing was initiated.

A summary of the endocrine values of patient 1–4 and clinical presentations is given in Table 1.

**Table 1:** Hormonal and clinical data of patients.

Age (years; months)	Clinical presentation	T (ng/dL)	FSH (IU/L)	LH (IU/L)	ACTH (pg/mL)	Cortisol ng/mL	Other investigations
**Patient 1** At birth	Ambigous genitalia, phallus 1.3 cm, small vagina	**20**	**13.2** (ref: <11)	3.9	54 (ref: <60)	26, ACTH test: Basal: 8.2, Stimulated: 54.3	
10;7	Testicular volume 3–4 cc, Tanner stage G2	82.6, after hCG stimulation: 242	8.8	1.03			Inhibin B: 19 ng/L
11;3		135.8	**16.5**	2.27			Inhibin B <10 ng/L
11;11		162	**20.9**	5.2			
15;8	Genital appearance: Tanner stage G4/ PH6, testes volume 4–6 cc	508 (under suppl. with 37.5 mg testosterone gel)	**18.2**	7.6	39.2	154	Height: 180.5 cm weight: 64.3 kg
16;7			**18.7**	9.4		ACTH test: Basal: 182, Stimulated: 319	
17;3			**33.1**	**19.4**			Inhibin B 11 ng/L
18;2		383 without testosterone supplementation	**56.1**	**44.3**			TESE
**Patient 2** At birth	Ambigous genitalia, rugated labioscrotal folds, partial labioscrotal fusion, phallus 1.3 cm, hypospadias, short urogenital sinus	< 1 (ref: 2.8–362.9). After hCG stimulation: 8	7.4	<0.5		ACTH test; Basal: 11 (ref: 9.24–86.8). Stimulated: 187 (ref: 54–718)	hCG stimulation: basal: androstenedione **6 ng/dL** (ref: 11.4–60 ng/dL). Stimulated: 40 ng/dl; DHT < 1 ng/dL (ref: 2.8–11.6 ng/dL). Stimulated: **< 1 ng/dL**
8.4	Inguinal hernia						
**Patient 3** At birth	Bipartite scrotum, phallus 2 cm with chordae, hypospadias scrotalis, testes descended	187					
0;3.5		hCG test: Basal: 232, Stimulated: 735	3.7	2.6			Inhibin B: 144 ng/L hCG test: DHT basal:80 ng/dL, stimulated: 469 ng/dL
12	Short penis, testes volume 4/2 cc	119	9	2.4			Inhibin B: 36.2 ng/L
14;7	Pubertal stage Tanner PH4/G4, testicular volume: 6/8 cc	297	**16.2** (ref:<12.4)	3.4	69.4	170	
16		344	**27.1**	7.5	45.9	96	Inhibin B: 15 ng/L
**Patient 4** 14;5	Primary amenorrhea, small uterus of prepubertal size, pubertal stage Tanner PH1/B1, small dysgenetic gonads with rudimentary testicular tissue, gonadectomy and estradiol supplementation	**16**	**90.7**	**31.6**	25.16	38.5	Inhibin B:<10 ng/L
15;10	Pubertal development Tanner B4, uterus 4 cm, no menarche		**81.4**	**49.3**			Estradiol 28.5 ng/L (under supplementation)

Endocrine values outside the reference ranges are given in bold. T = testosterone

### Sequence analysis of the *NR5A1* gene

Genomic DNA was extracted from peripheral blood leukocytes using the Qiaquick DNA kit (Qiagen, Hilden, Germany). Exons 1–7 of *NR5A1* including the exon-intron boundaries were amplified and sequenced by direct cycle sequencing using the BigDye Terminator v3.1 Cycle Sequencing Kit (Applied Biosystems) and a 3130 Genetic Analyser (Applied Biosystems).

### Exome capture and sequencing

Exome capture and sequencing were performed as described previously [42]. The exomes of both affected children (P3; P4) and their parents were sequenced as 100 bp paired ends on a HiSeq2000 System (Illumina) generating between 8.66 and 14.99 Gb of sequence data with an average read depth of 103 x to185 x on target regions.

### Plasmids

The c-myc-tagged *NR5A1* expression plasmid and the *STAR* and *AMH-luciferase* reporter genes were kindly provided by Dr. Yukihiro Hasegawa, Tokyo and have been described previously [19]. The mutation c.118A>C and c.51_65del were generated by a two-step mediated PCR. In the first step two intermediate PCR products were amplified using *NR5A1* wt vector as template and PCR primers CMV_forw: 5’-gatccggtactagaggaactgaaaaac-3’ and NR5A1_a118c_AS: 5’- tgttgttctgcaccgGgcgcttgaagaagcc-3’ for PCR 1 and primers NR5A1_a118c_S: 5’- ggcttcttcaagcgcCcggtgcagaacaaca-3’ and SF1_Ex4intAS_RP: 5’- gcagcacgtagtccggtg-3’ for PCR 2 or mutation c.118A>C and primers CMV_forw and NR5A1_del51_65_AS: 5’- tccgtagtggtagccgcacacggggc-3’ for PCR 1 and primers NR5A1_del51_65_S: 5’- gccccgtgtgcggctaccactacgga-3’ and SF1_Ex4intAS_RPfor PCR 2 for mutation c.51_65del. The resulting fragments were gel purified, annealed and amplified in a third PCR using primer pair CMV_forw and SF1_Ex4intAS_RP for both mutations. These amplicons were again purified and subcloned into the NR5A1 expression vector using the restriction sites *Eco*RI and *Afe*I for c.118A>C and *Eco*RI and *Bsr*GI for c.51_65del. The correct sequence was verified by Sanger sequencing.

### Cell culture and transient transfection assays

Transient transfection assays were performed in 24 well plates. 50.000 HeLa cells/ well were grown over night in Dulbecco’s Modified Eagle Medium/ Nutrient Mixture F-12 Ham (DMEM, Sigma, Aldrich) supplemented with 10% foetal bovine serum (FBS) in 5% CO_2_ at 37°C. Cells were transfected using 30 ng of wt or mutant *NR5A1* construct or empty vector, 200 ng of the respective *AMH*- or *STAR*-reporter plasmid and 10 ng of pGL4.74 Renilla luciferase (Promega) to normalise for transfection efficiency and 0.72 μL FuGene HD (Promega). After 24 hours luciferase activity was determined using the Dual Luciferase Assay System (Promega). Experiments were performed in triplicates and at least 3 independent experiments were carried out. For comparison of experiments performed at different days, activity of wt-NR5A1 was set 100%. HeLa cells (DSMZ no. ACC 57) were obtained from Leibnitz-Institute DSMZ, Göttingen (Deutsche Sammlung von Mikroorganismen und Zellkulturen GmbH).

### Immunoblotting

To analyse protein expression HeLa cells were transfected and cultured as described before. After a 24 hours incubation they were washed twice with phosphate buffered saline (PBS) and lysed with 100 μl/well ice-cold Mammalian Protein Extraction Reagent (M-Per, ThermoScientific) including protease inhibitor cocktail (cOmplete Ultra Tablets, Mini, EDTA-free, Roche). The cells were incubated on a shaker on ice for 5 minutes, scraped and homogenized using Qiashredder columns. The protein extracts were diluted 1:1 with 2x sample buffer (4% SDS, 20% glycerol, 120 mM Tris-HCl, 200 mM dithiothreitol, 0.02% bromophenol blue, pH 6,8) and denaturated for 10 minutes at 95°C. The proteins were separated by SDS-Page using a 10% discontinuous TGX Stain Free polyacrylamide gel (BioRad). After UV-activation of the gel proteins were blotted onto a nitrocellulose membrane (Bio-Rad) and total protein was visualized by ChemiDoc Touch Imaging System (BioRad) The membrane was blocked in 5% Skim Milk (BD) in PBS with 0.1% Tween-20 (Sigma-Aldrich) over night at 4°C. Myc-tagged SF-1 was detected with the primary antibody anti-c-myc-peroxidase (clone 9E10, Roche) in a 1:1000 dilution for one hour at room temperature. Specific bands were detected by ChemiDoc Touch Imaging System (BioRad) or Fusion SL chemiluminescence imaging system (Vilber Lourmat) using Western Lightning Plus ECL (Perkin Elmer) as substrate.

### Nuclear extracts and electrophoretic mobility shift assay

300.000 HeLa cells per well were cultured in 6 well plates as described before and transfected with either 960 ng/well of the WT, mutant or empty *NR5A1* expression vector using 2.88 μl FuGene HD (Promega)/well. 24 hours later nuclear and cytoplasmic extracts were isolated using NE-PER Nuclear and Cytoplasmic Extraction Reagents and Halt Protease Inhibitor Single-Use Cocktail EDTA-Free (both Thermo Scientific). Electrophoretic mobility shift assays (EMSA) were performed using Light Shift Chemiluminescent EMSA Kit (Thermo Scientific) according to manufacturer’s instructions. Nuclear extracts were incubated with 5’-biotin labelled oligonucleotides containing a SF-1 responsive element of the mouse *AMH* promoter without or with either a 200 fold excess of unlabelled target DNA or unlabelled mutated target DNA. Samples were separated by non-denaturating polyacrylamide gel electrophoresis, blotted onto a nylon membrane (Hybond-Nx, Amersham Pharmacia Biotech), UV-crosslinked and detected using Fusion SL chemiluminescence imaging system (Vilber Lourmat). All sequences of oligonucleotides comprising the SF-1 responsive element were previously described [43] and are presented in S1 Table.

### Bioinformatics

The newly identified variants were annotated using ANNOVAR [44].

Allele frequencies of these variants were estimated using the data of the exome sequencing project (6500 samples) (Exome Variant Server, NHLBI GO Exome Sequencing Project (ESP), Seattle, WA (URL:http://evs.gs.washington.edu/EVS/), the Exome Aggregation Consortium Browser [45] and of the 1000 Genomes Project (phase3v5, 2483 unrelated samples [46].

## Results

### Genetic analysis

**Fig 1:**
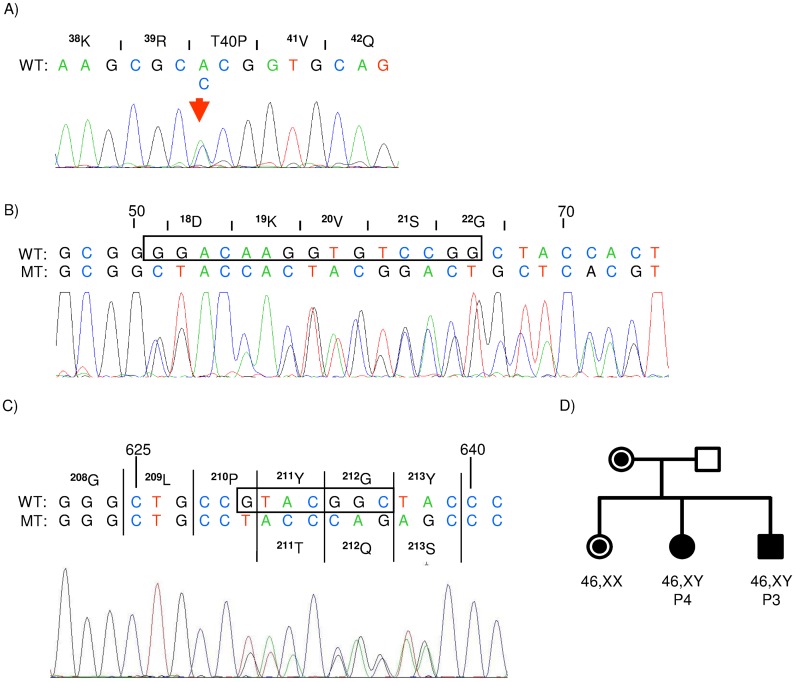
Electropherogram of *NR5A1* mutations. Electropherogram of the heterozygous c.118A>C mutation in patient 1 (A), the nonframeshift deletion c.51_65delGGACAAGGTGTCCGG in patient 2 (B), the frame-shift deletion c.630_636delGTACGGC in patient 3 (C) and the pedigree of the family of patient 3 and 4 (D). Circles denote phenotypic females, squares denote phenotypic males. Filled squares and circles correspond to a DSD condition, dots to a carrier status.

**Fig 2:**
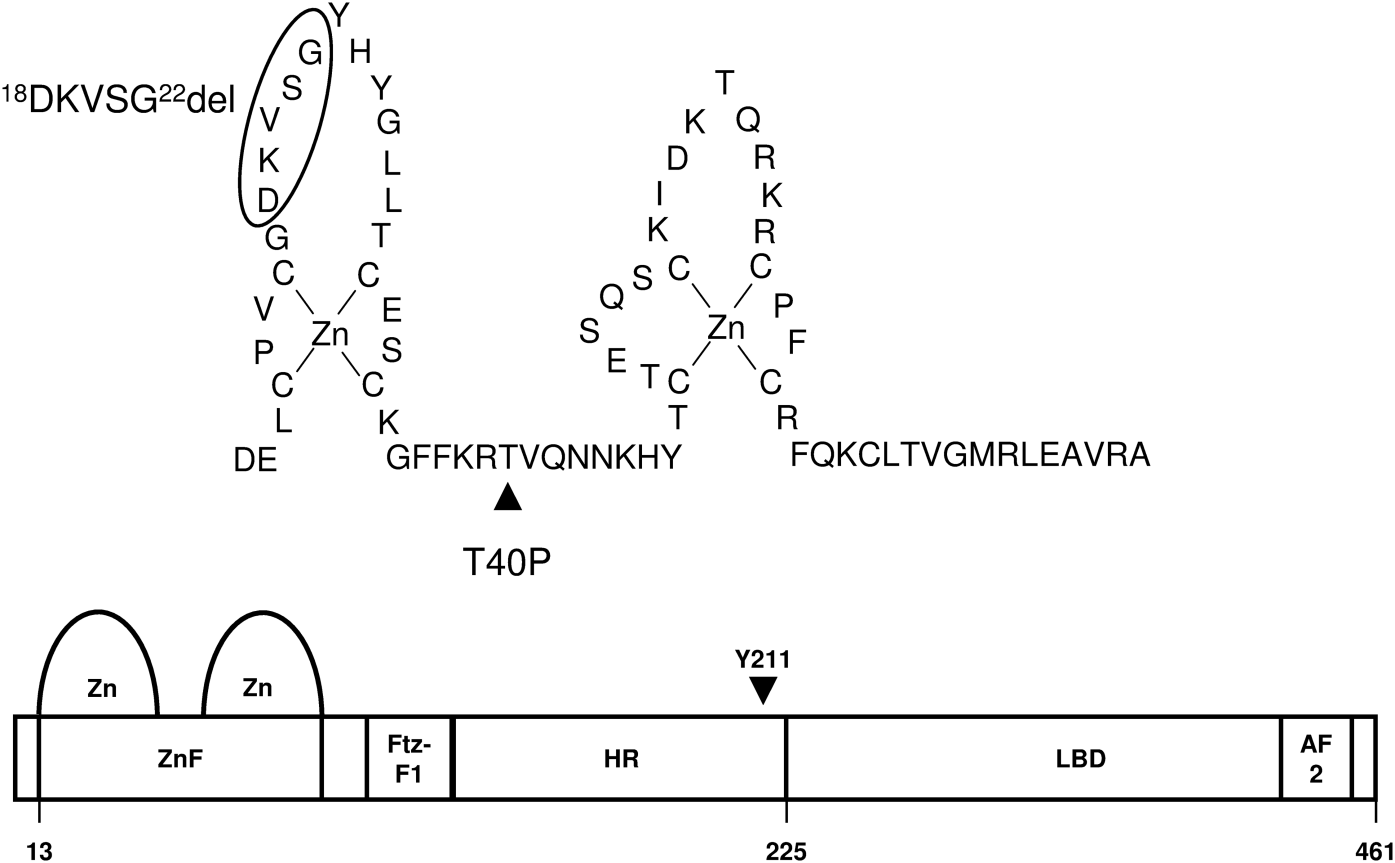
Schematic view of NR5A1. Scheme of the DNA-binding domain containing the zinc finger domain (ZnF) and the Ftz-F1 box, the hinge region (HR) and the ligand binding domain (LBD) with activation function 2 (AF2) of NR5A1. Positions of the mutation p.T40P, p.D18_G22del and amino acid Y211 are indicated.

Sequence analysis of the *NR5A1* gene of patient 1 revealed a heterozygous c.118A>C missense mutation in exon 3 leading to a p.T40P substitution in the DNA binding domain. In patient 2 a heterozygous small nonframeshift deletion (c.51_65del) in exon 2 of the *NR5A1* gene was identified (Fig 1) leading to a deletion of five amino acids (^18^DKVSG^22^) within the loop of the first zinc finger of the protein (Fig 2).

Exome sequencing of the family of patient 3 and patient 4 revealed a common heterozygous 7 bp deletion (c.630-636del) in exon 4 of *NR5A1* in both patients and their mother. This mutation leads to a frameshift in the hinge region at amino acid residue 211 (p.Y211Tfs*83) and was later also found in their elder 46,XX sister (Fig 1). Patient 4 developed a complete gonadal dysgenesis, while her younger brother developed a much milder partial gonadal dysgenesis. Therefore, we analysed whether both children also inherited the same wt-*NR5A1* allele from their father. Nearest informative SNPs are rs2281923 and rs8913231 in *OLFML2A* and rs10818907 and rs104486 in the *LHX2* gene located 0.3 Mb upstream and 0.48 Mb downstream from the microdeletion. These markers reveal that both siblings most likely have inherited the same wt-*NR5A1* allele from the father (S5 Fig). We also searched for additional deleterious mutations in the exome of the girl but not the boy affecting genes with a possible function in gonadal development or androgen pathways. A good candidate found only in the girl and her mother was a heterozygous missense mutation in *TBX2* (NM_005994.3:c.641A>G; p.N214S).

### Functional studies

#### Reporter gene assays

**Fig 3:**
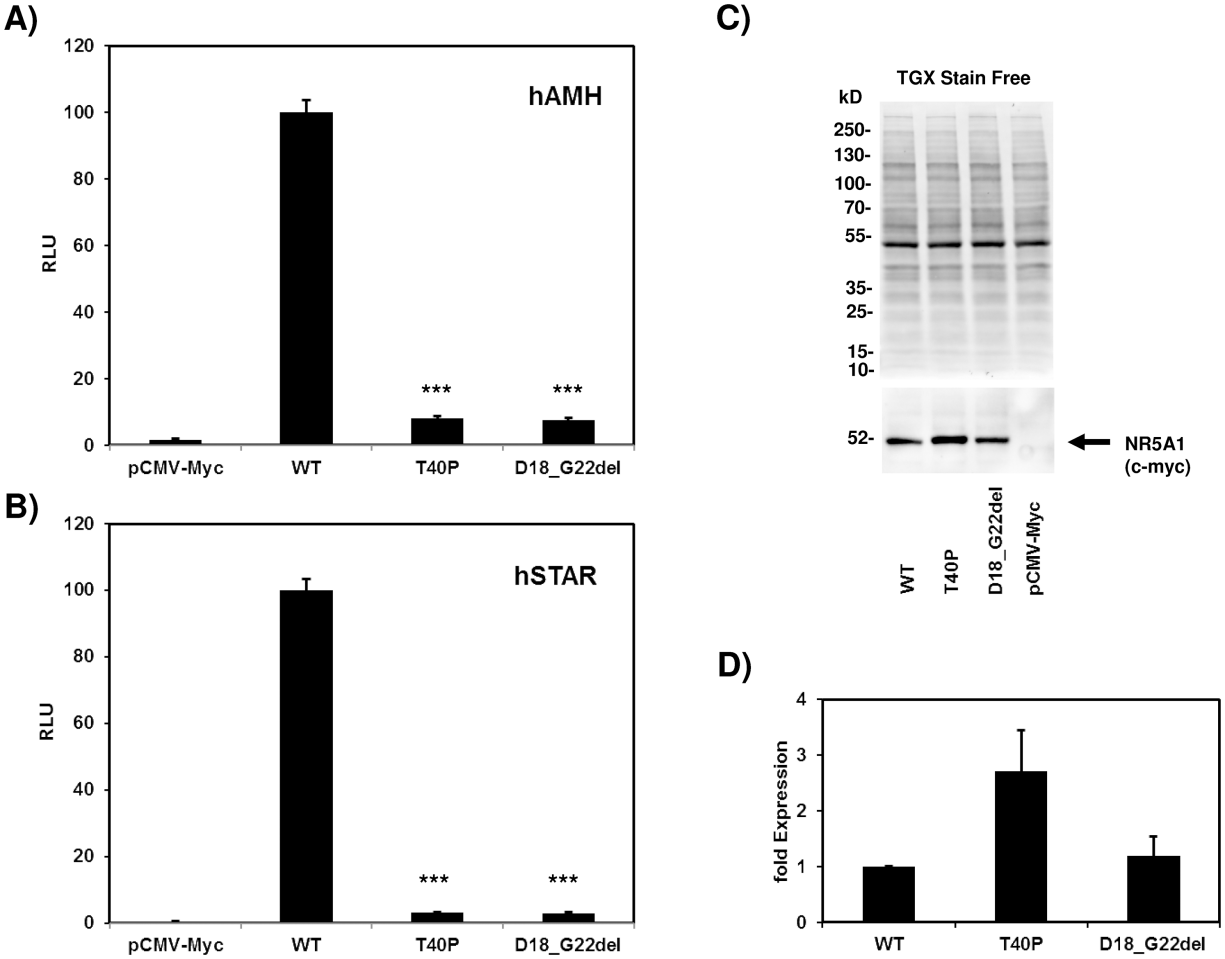
Transactivation of the human *AMH*- and *STAR*-promoter. The transactivation capacity of mutant NR5A1-T40P and NR5A1-^18^DKVSG^22^del were compared to wt-NR5A1 using human *AMH-* (A) and *STAR*-promoter (B) containing reporter genes. The empty pCMV-Myc vector represents background activity. Wild type (WT) activity was set 100%. RLU = relative luciferase activity. Error bars represent standard deviations, ***P = <0.001, t-test comparison of WT and mutant. C: Immunoblot detection of myc-tagged NR5A1 proteins. Equal loadings were verified by detection of total proteins using the TGX Stain Free system (BioRad). D: statistical analysis of 3 western blots of three different experiments showing an approximately equal protein accumulation of NR5A1-WT and mutant ^18^DKVSG^22^del, while mutant T40P showed an 2–3 fold enhanced accumulation. Images were quantified and normalized to total protein using Image Lab 5.2.1 (BioRad).

To study the impact of both zinc finger mutations to the transactivation capacity of SF-1 *in-vitro*, HeLa cells were co-transfected with the mutant or wild type NR5A1 expression construct and a NR5A1 responsive *STAR*- or *AMH*-reporter gene and transient expression assays were carried out. Both SF-1 mutants display an extremely reduced transactivation capacity on both reporter genes (below 10% on the *AMH* promoter, below 5% on the *STAR* promoter) compared to the wild type (Fig 3). Immunoblots showed a similar accumulation of mutant NR5A1-^18^DKVSG^22^del and wild type SF-1 proteins, while NR5A1-T40P showed an enhanced protein accumulation.

#### Electrophoretic mobility shift assays (EMSA)

**Fig 4:**
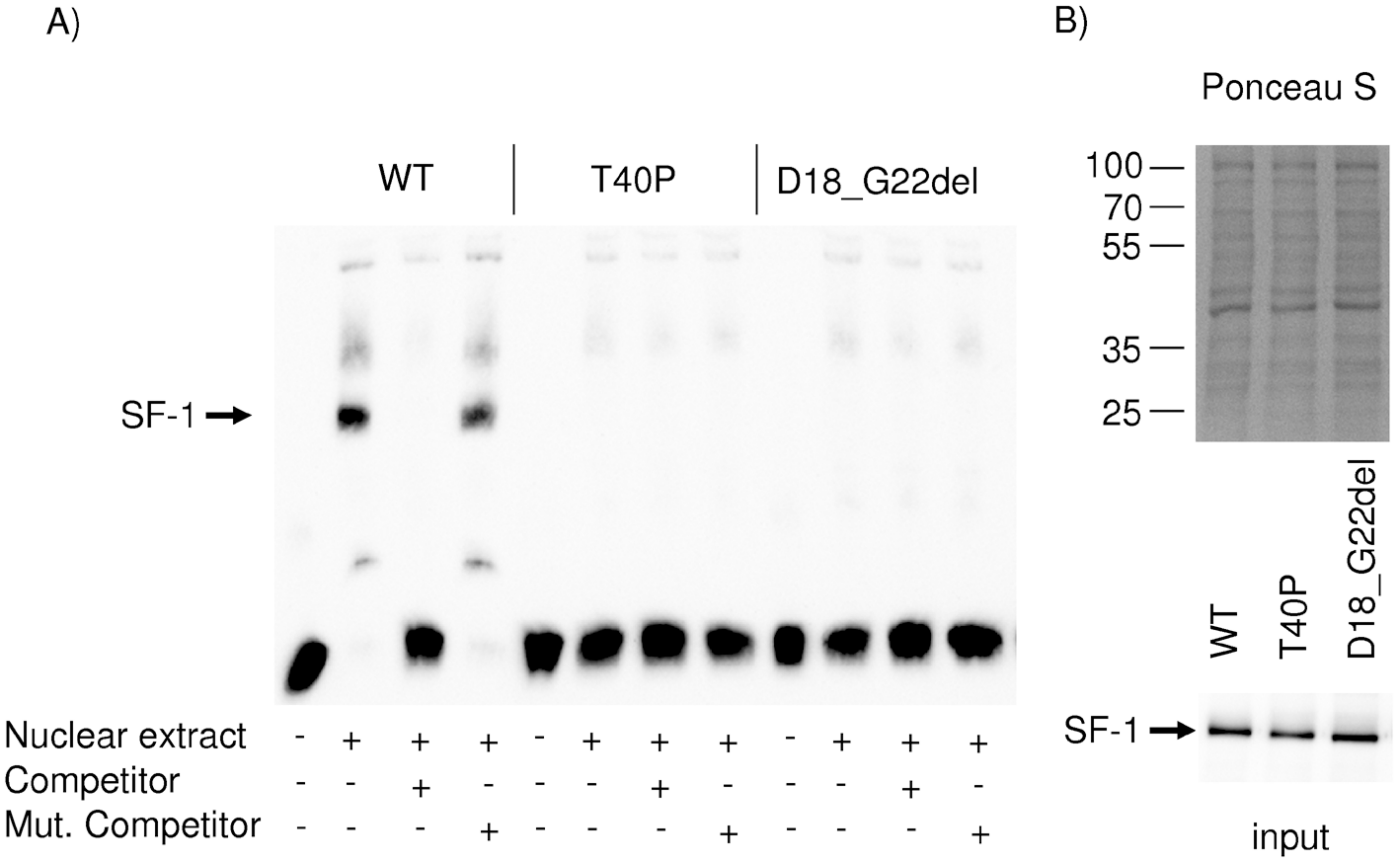
Electrophoretic mobility shift assay of NR5A1 mutants. A) DNA-binding capability of mutant NR5A1-T40P and NR5A1-^18^DKVSG^22^del were compared to wt-NR5A1 using a SF-1 responsive element of the mouse *Amh* promoter. Nuclear extracts containing WT-SF1 shifted the labelled probe (arrow). A 200 fold excess of unlabelled competitor abolished the shifted signal, while a 200 fold excess of unlabelled mutated competitor rescues the shifted signal. Both mutant proteins have lost their DNA-binding capability. B) Aliquots of the nuclear extracts used in EMSA were separated by SDS gel electrophoresis, transferred to a nitrocellulose membrane and stained with Ponceau S and anti-myc antibodies to verify equal SF-1 protein concentrations.

To assess the DNA binding ability of the SF-1 proteins mutated in the DNA-binding domain electrophoretic mobility shift assays were carried out using nuclear extracts of transfected HeLa cells and a SF-1 responsive element of the mouse *Amh* promoter. A complete loss of the DNA binding ability of mutant NR5A1-T40P as well as mutant NR5A1-^18^DKVSG^22^del could be observed. Aliquots of the nuclear extracts were separated by SDS-Page and blotted onto a nitrocellulose membrane. Staining with Ponceau S and an antibody against c-myc proved equal SF-1 protein concentrations used in EMSA (Fig 4).

### Bioinformatic analysis

All three *NR5A1* mutations and the *TBX2* mutation were novel and are neither listed in the database of the exome sequencing project ESP6500 or in the Exome Aggregation Consortium Browser nor the 1000 genomes phase 3 variant set, dbSNP146 or the Human Gene Mutation Database (HGMD). For missense mutations NR5A1 p.T40P and TBX2 p.N214S all pathogenicity prediction algorithms i.e. SIFT, PolyPhen2, LRT, MutationTaster or FATHMM, predict these mutations as deleterious, probably damaging or disease causing. The newly invented scaled Combined Annotation Dependent Depletion (CADD) scores (V1.3) [47] are 26.8 for NR5A1 p.T40P and 23.7 for TBX2 p.N214S and indicate deleterious mutations. These predictions are not available for frameshift mutations or nonframeshift deletions like NR5A1 p.Y211Tfsx83 or p.^18^D_^22^Gdel.

## Discussion

Today more than 100 different mutations in the *NR5A1* gene are listed in the Human Gene Mutation Database (HGMD) and have been described mostly associated with 46,XY DSD, but also associated with hypospadias, cryptorchidism, infertility or anorchia. In 46,XX individuals, mostly in mothers or sisters of 46,XY DSD patients an association to premature ovarian insufficiency (POI) have been observed [2]. Recently, also an association of the recurrent heterozygous NR5A1 mutation p.R92W with 46,XX testicular/ovotesticular DSD has been independently reported by three groups [25–27]. The wide spectrum of phenotypic associations shows the challenge of a genotype phenotype correlation in patients with *NR5A1* mutations. So far almost all *NR5A1* mutations associated with DSD are described as heterozygous mutations. Mice with a homozygous targeted deletion of the *Nr5a1* gene displayed a lack of adrenal glands and gonadal agenesis and died at postnatal day 8 because of the lack of corticosteroids [4, 48]. Only three homozygous mutations have been described so far [23, 29, 32, 49, 50]. The homozygous mutation R92Q is associated with 46,XY DSD and primary adrenal insufficiency in 46,XX and 46,XY patients. The homozygous mutation D293N has been found in a 46,XX patient with primary ovarian failure and a 46,XY patient with complete gonadal dysgenesis. The third, a homozygous R103Q mutation, is associated with 46,XY DSD and asplenia. All naturally occurring homozygous mutations showed only a partial loss of function in transactivation assays, which may preserve fertility of the transmitting fathers in these cases and points to the dosage sensitivity of NR5A1 mutations.

The mutations of P1 and P2 lie within the N-terminal zinc finger of the highly conserved DNA-binding domain and have not been described before. The predicted deleterious, probably damaging or disease causing nature of mutation p.T40P and the disruptive effect of the nonframeshift deletion of five amino acids within the loop of the first zinc finger in mutation p.D18_G22del was confirmed experimentally by a severe loss of DNA-binding or transactivation capacity as demonstrated by EMSA and two different NR5A1-dependent reporter gene assays using fragments of the human *STAR* and *AMH* promoter. The severe functional deficit of both mutant proteins may explain the phenotype of ambiguous genitalia observed after birth but hardly predicts pubertal outcome or gender assignment. Promoters in NR5A1 target genes show several variations of the consensus binding site (Py)CA AGGTCA and it has been shown for the NR5A1 mutant G35E that mutations in the DNA-binding domain can lead to a differential binding and activation of these target genes [51]. Amino acid T40 lies within the recognition helix also referred to as the “P-box”. The neighbouring residues R39 and K38 are forming hydrogen bonds to the hormone response element (HRE) [52] and intramolecular ionic and hydrophobic bonds to T40. Molecular modelling revealed that these intramolecular bonds are altered or lost in the mutant p.T40P and may contribute to a loss of DNA recognition.

Two natural NR5A1 mutations adjacent to T40 have been described, p.R39P and p.V41P. Both are associated to 46,XY girls with clitoromegaly and primary amenorrhea and low testosterone values [53, 54]. Patient 1 (p.T40P) presented at birth also with a small vagina and a low testosterone value and was at first assigned to a female sex. The presence of testicular tissue with germ cells and a relevant, although subnormal, testosterone synthesis and absence of Müllerian structures led to a reassignment of P1 into male gender. At puberty a spontaneous but subnormal rise in testosterone level lead to a progressive virilisation that was fostered by additional testosterone gel supplementation with good response. The drop of inhibin B and a severe rise in FSH level pointed to a progressive testicular dysfunction observed also in P3 and other patients with *NR5A1* mutations [20, 38, 55].

The reason for the high intra-familiar phenotypic variability observed between P3 and P4 and also in other families still remains elusive [24, 56]. Further mutations in NR5A1, cofactors of NR5A1, or in other genes of the sex development cascade have been discussed. In addition, the expression ratio of wt to mutant alleles in the patients must not be equal and may also rely on differences within the promoter region of the inherited wt allele. In the family described here, a difference in NR5A1 expression, due to different inherited paternal wt-alleles is unlikely, since the nearest available informative SNPs upstream and downstream the *NR5A1* microdeletion indicate a common paternal allele in P3 and P4. Interestingly, a very similar *NR5A1* deletion (c.630_637del; p.Y211Pfs*12) was found in another family with 3 affected members [56]. A grandfather with proximal hypospadias, who transmitted the mutation to two 46,XX girls of whom one suffered from premature ovarian failure (POF), while the other was unaffected and transmitted the mutation to her 46,XY son suffering also from hypospadias. We analysed the exome data for additional mutations occurring de novo in P4 or inherited in P4 from her mother, that might aggravate a more severe gonadal dysgenesis in P4. A good candidate is a heterozygote missense-mutation in the DNA-binding domain of *T-box factor 2* (*TBX2*) found in P4 and her mother. *TBX2* is a transcription factor expressed in the mouse at E13.5 in the interstitium as well as in the Wolffian and Müllerian ducts and may have a role in Leydig cell development, Wolffian duct differentiation or Müllerian duct regression [57].

Possible fertility proven by paternal transmission of *NR5A1* mutations in rare cases [56, 58] may indicate early intervention to preserve fertility. Semen analysis of P1 revealed azoospermia and testicular sperm extraction (TESE) and cryopreservation was considered to preserve fertility. Unfortunately, no sperms could be revealed in this case. If TESE and intracellular sperm injection (ICSI) are successful options in 46,XY DSD might be shown in the future.

Gonadal dysgenesis in the clinical context has been defined by the presence of Müllerian structures in 46,XY DSD. Mutations in *NR5A1* have been observed in men with infertility or hypospadias as well as in 46,XY individuals with ambiguous genitalia or anorchia, but also in 46,XY girls with primary amenorrhea with and without Müllerian structures. Very recently it has been shown that this spectrum of DSD phenotypes is extended also to 46,XX ovotesticular DSD (OTDSD) and 46,XX males with testicular DSD (TDSD) [25, 26]. Moreover associated mutations show incomplete penetrance and can be inherited by unaffected fathers or mothers in 46,XY DSD, but also in familiar cases of 46,XX OTDSD/TDSD [10, 25, 26, 55]. A model is emerging in which NR5A1 can act as a switch in sex development and mutations in NR5A1 either lead to an insufficient upregulation of *SOX9* expression in 46,XY DSD or an insufficient repression of *SOX9* in 46,XX OTDSD/TDSD [25–27]. Also, the clinical diagnosis of gonadal dysgenesis has to be revised.

In contrast to mice, where Nr5a1 is sexually dimorphic expressed in somatic cells at the early stage of testis and ovary development human NR5A1 is upregulated in the undifferentiated gonad, maintained in the period of fetal development in both sexes and may contribute to the development of testes and ovaries [26, 59]. These interspecies specific differences have been also shown by clustered regulatory interspaced short palindromic repeats (CRISPR)/Cas9 induced genome editing and introduction of p.R92W into mice [60, 61].

Adrenal insufficiency associated to heterozygous and homozygous mutations in *NR5A1* have been detected in three 46,XY DSD patients as well as in two 46,XX girls. Interestingly, the same homozygous R92Q mutation was detected in a 46,XY DSD patient and a 46,XX girl from two different families [29, 32]. As mentioned above, this mutation showed a reduced DNA-binding and reporter gene transactivation, while the heterozygous NR5A1 mutations p.G35E and p.R255L have lost their ability to activate reporters or bind to DNA [28, 30]. The third heterozygous NR5A1 mutation associated to adrenal insufficiency and 46,XY DSD is E7X and is considered as a loss of function mutation [31]. These rare observations implicate that adrenal insufficiency is associated to more severe *NR5A1* mutations. Therefore we measured ACTH and cortisol values or performed ACTH stimulation tests and monitored basal and stimulated plasma steroid values. Our patients showed normal cortisol values or a good response to ACTH and no evidence for adrenal insufficiency. Nevertheless, adrenal function should be followed in carriers of *NR5A1* mutations, because additional factors that contribute to phenotype in the rare cases described are unknown and adrenal insufficiency may develop over time.

## Supporting information

S1 Table Sequences of oligonucleotides used in EMSA. The oligonucleotides contain either a core SF-1 binding motif of the mouse AMH promoter (mMIS) or a mutated SF-1 binding motif mMIS_mut. The SF-1 binding motif is highlighted in bold. Oligonucleotide sequences have been described previously [43].
(DOCX)

S1 Fig Laparoscopic examination of patient 2. Retracted testis-like right and left gonads out of the inguinal channels, respectively. No epididymis or ductus deferens could be detected on either gonad.
(TIF)

S2 Fig Biopsy of the gonad of patient 2 at the age 8 years. Immature Sertoli-cell-only tubules with small or absent lumina and reduced tubular diameter. No Leydig cells were detected in the interstitium.
(JPG)

S3 Fig Dysgenetic gonad of P4. CYP17A1 staining revealed clusters of vacuolated Leydig-cell-like cells in fibrotic stroma of the gonad A) overview 40x, B) 400x magnification. A very low testosterone value of 16 ng/dl in P4 indicates non-sufficient testosterone synthesis despite high CYP17A1 expression. C) Other parts of the gonad reveal epididymal and tubular tissue (HE stain).
(TIF)

S4 Fig Sonography of the uterus of P4. A) before and B) after estrogen treatment.
(TIF)

S5 Fig Both siblings carry the same *NR5A1* alleles. Nearest informative SNPs in the exome data are located 0.3 Mb upstream and 0.48 Mb downstream of the micro deletion and revealed that both siblings carry the same *LHX2* and *OLFML2A* alleles from the father and, therefore most likely, also the same *NR5A1* alleles from the mother as well as the father.
(TIF)
