# Supplementary material for: New NR5A1 mutations and phenotypic variations of gonadal dysgenesis
Source: PLoS One. 2017 May 1;12(5):e0176720. doi: 10.1371/journal.pone.0176720 (PMC5411087; doi:10.1371/journal.pone.0176720)
Supplement: S1 Table — The oligonucleotides contain either a core SF-1 binding motif of the mouse AMH promoter (mMIS) or a mutated SF-1 binding motif mMIS_mut. The SF-1 binding motif is highlighted in bold. Oligonucleotide sequences have been described previously [43]. (DOCX) [file pone.0176720.s001.docx]

**S1 Table: Sequences of oligonucleotides used in EMSA**

| SF1_EMSA_mMISs | 5’-GCC AGG CAC TGT CCC **CCA AGG TCA** CCT TTG GTG TTG ATA-3’ |
| --- | --- |
| SF1_EMSA_mMISa | 5’-TAT CAA CAC CAA AGG TGA CCT TGG GGG ACA GTG CCT GGC-3’ |
| 5Bio-SF1_EMSA_mMISs | 5’-Biotin-GCC AGG CAC TGT CCC **CCA AGG TCA** CCT TTG GTG TTG ATA-3’ |
| mMIS_mutS_EMSA | 5’-GCC AGG CAC TGT CCC **CCA ATT TCA** CCT TTG GTG TTG ATA-3’ |
| mMIS_mutA_EMSA | 5’-TAT CAA CAC CAA AGG TGA AAT TGG GGG ACA GTG CCT GGC-3’ |
| 5Bio-mMIS_mutS_EMSA | 5’-Biotin-GCC AGG CAC TGT CCC **CCA ATT TCA** CCT TTG GTG TTG ATA-3’ |

The oligonucleotides contain either a core SF-1 binding motif of the mouse AMH promoter (mMIS) or a mutated SF-1 binding motif mMIS_mut. The SF-1 binding motif is highlighted in bold. Oligonucleotide sequences have been described in: Campbell LA, Faivre EJ, Show MD, Ingraham JG, Flinders J, Gross JD, Ingraham HA: Decreased recognition of SUMO-sensitive target genes following modification of SF-1 (NR5A1). Mol Cell Biol 28:7476-7486 (2008).
