# Supplementary figures and images for: New NR5A1 mutations and phenotypic variations of gonadal dysgenesis
Source: PLoS One. 2017 May 1;12(5):e0176720. doi: 10.1371/journal.pone.0176720 (PMC5411087; doi:10.1371/journal.pone.0176720)

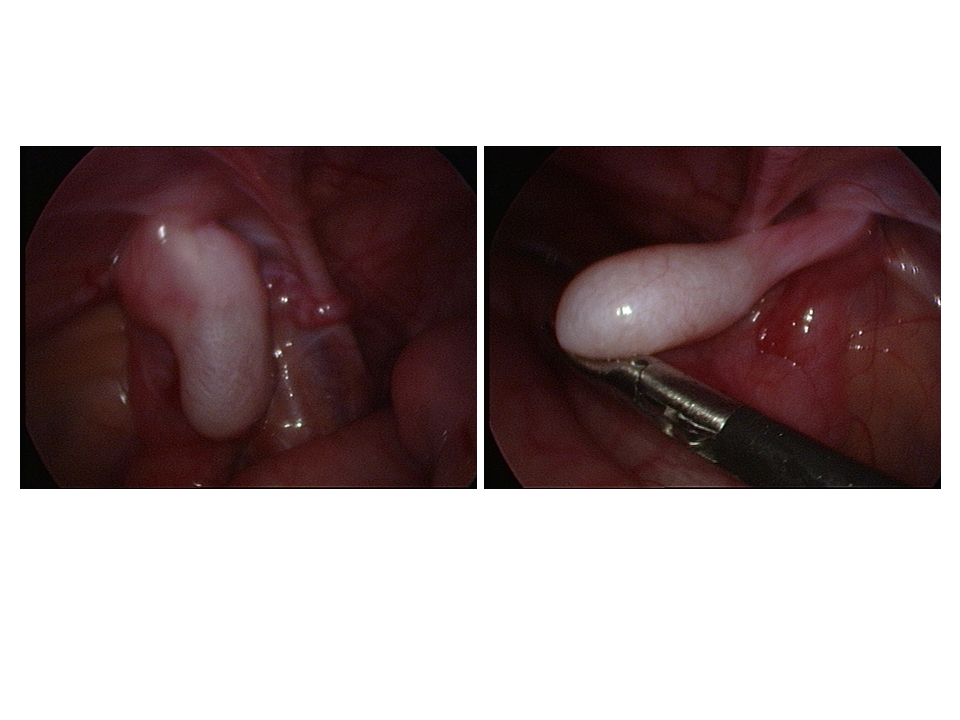

Supplement: S1 Fig — Retracted testis-like right and left gonads out of the inguinal channels, respectively. No epididymis or ductus deferens could be detected on either gonad. (TIF) [file pone.0176720.s002.tif]

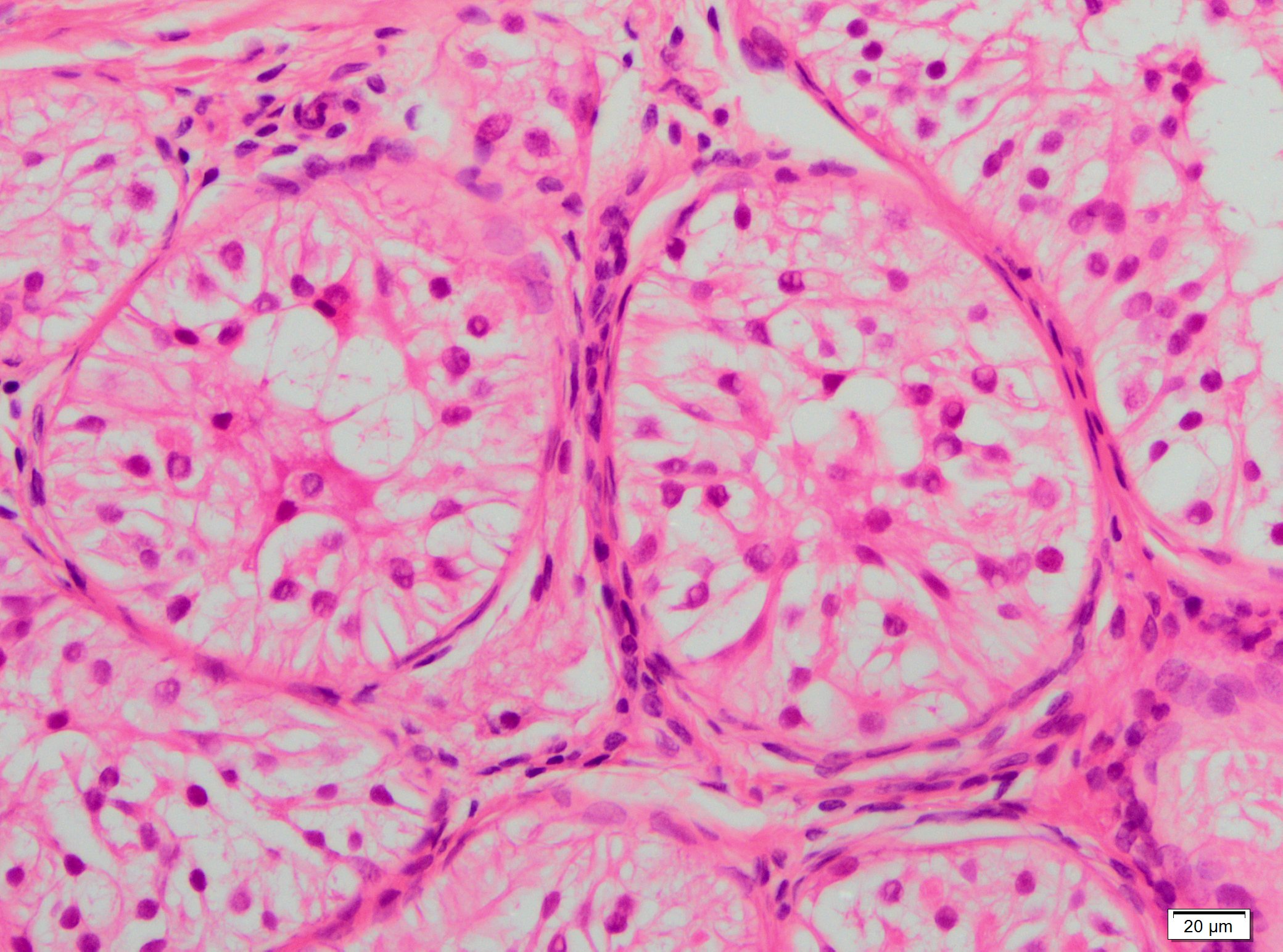

Supplement: S2 Fig — Immature Sertoli-cell-only tubules with small or absent lumina and reduced tubular diameter. No Leydig cells were detected in the interstitium. (JPG) [file pone.0176720.s003.jpg]

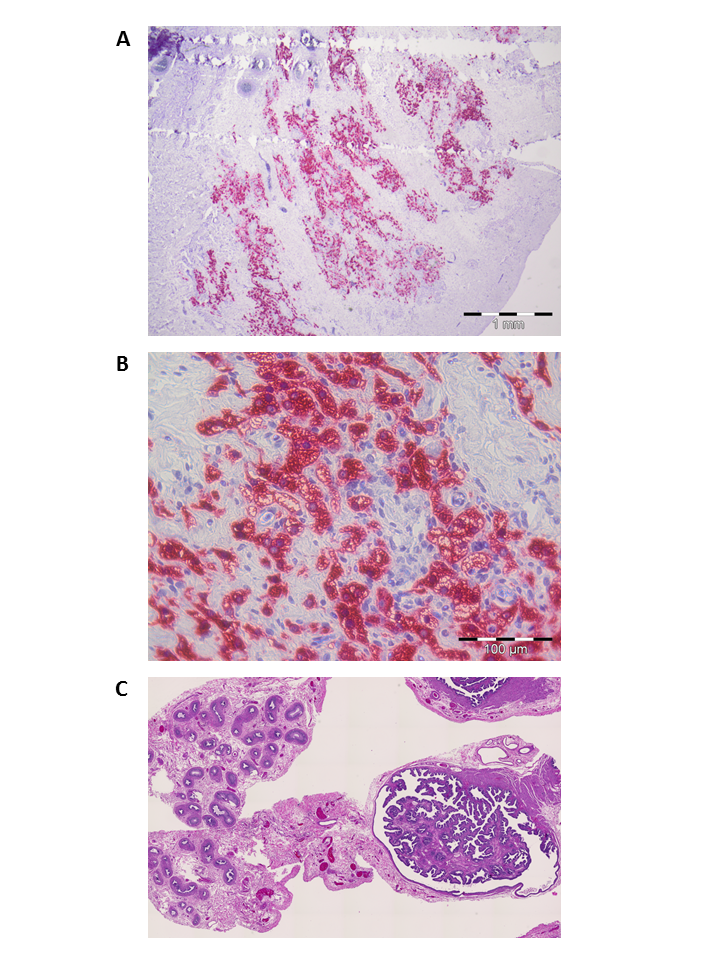

Supplement: S3 Fig — CYP17A1 staining revealed clusters of vacuolated Leydig-cell-like cells in fibrotic stroma of the gonad A) overview 40x, B) 400x magnification. A very low testosterone value of 16 ng/dl in P4 indicates non-sufficient testosterone synthesis despite high CYP17A1 expression. C) Other parts of the gonad reveal epididymal and tubular tissue (HE stain). (TIF) [file pone.0176720.s004.tif]

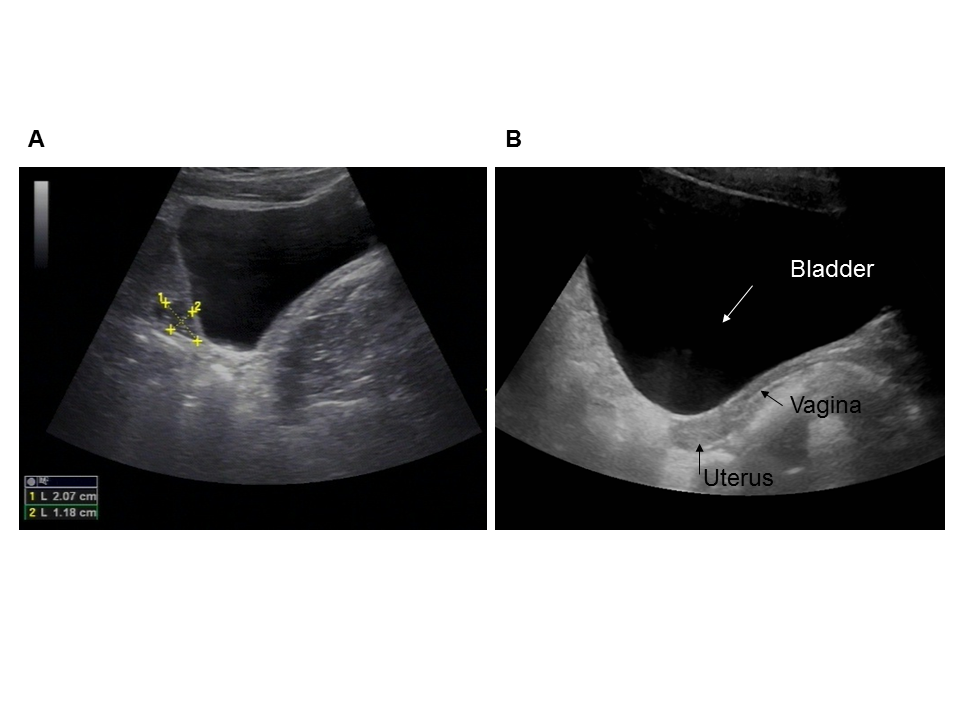

Supplement: S4 Fig — A) before and B) after estrogen treatment. (TIF) [file pone.0176720.s005.tif]

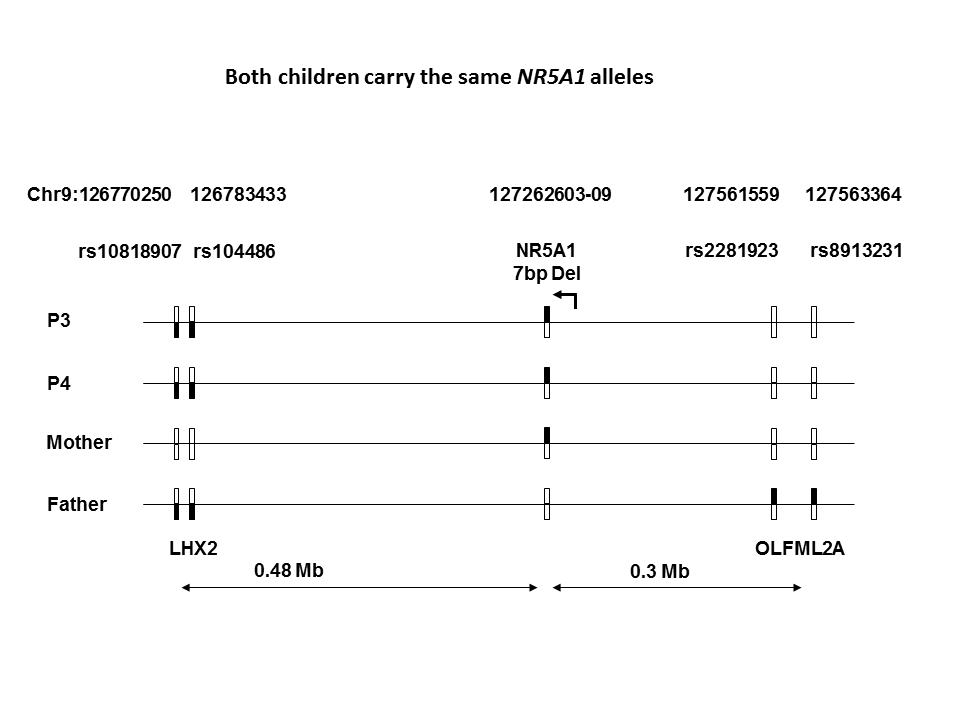

Supplement: S5 Fig — Nearest informative SNPs in the exome data are located 0.3 Mb upstream and 0.48 Mb downstream of the micro deletion and revealed that both siblings carry the same LHX2 and OLFML2A alleles from the father and, therefore most likely, also the same NR5A1 alleles from the mother as well as the father. (TIF) [file pone.0176720.s006.tif]
